# Supplementary material for: Conditional chemoconnectomics (cCCTomics) as a strategy for efficient and conditional targeting of chemical transmission
Source: eLife. 2024 Apr 30;12:RP91927. doi: 10.7554/eLife.91927 (PMC11060718; doi:10.7554/eLife.91927)
Supplement: Figure 3—source data 3. [file elife-91927-fig3-data3.zip › Figure 3 source data3 part 2.zip!/Figure 3-Source data 3-part2/1062-13-CRISPResso_Report_jt28fhr9.zip!/CRISPResso_on_1062_15/3b.gcaggaaatgagctacctac.Insertion_deletion_substitutions_size_hist.pdf]

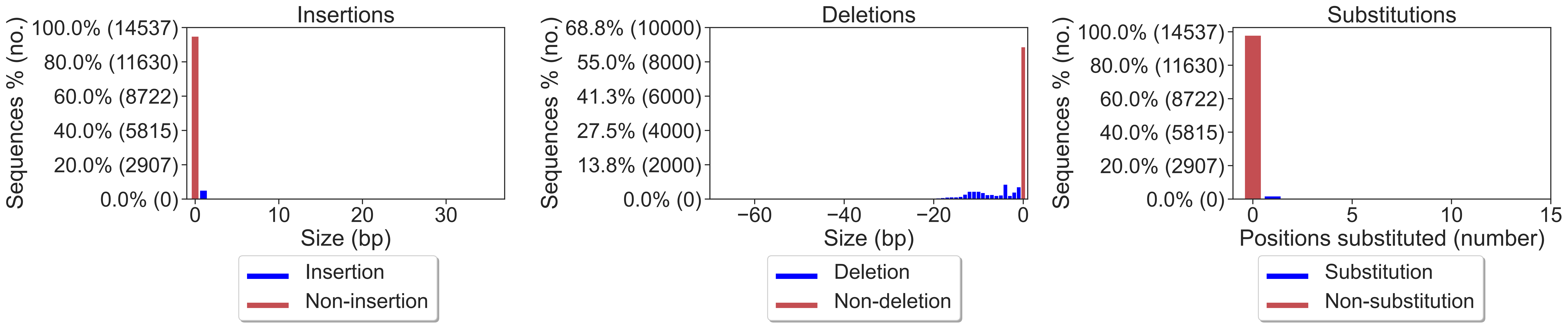

Insertions
Sequences % (no.)
100.0% (14537)
80.0% (11630)
60.0% (8722)
40.0% (5815)
20.0% (2907)
0.0% (0)
0
10
20
30
Size (bp)
Insertion
Non-insertion
Deletions
Sequences % (no.)
68.8% (10000)
55.0% (8000)
41.3% (6000)
27.5% (4000)
13.8% (2000)
0.0% (0)
−60
−40
−20
0
Size (bp)
Deletion
Non-deletion
Substitutions
Sequences % (no.)
100.0% (14537)
80.0% (11630)
60.0% (8722)
40.0% (5815)
20.0% (2907)
0.0% (0)
0
5
10
15
Positions substituted (number)
Substitution
Non-substitution
